# Association between elasticity of tissue and pain pressure threshold in the tender points present in subjects with fibromyalgia: a cross-sectional study

**DOI:** 10.1038/s41598-023-49550-y

**Published:** 2023-12-12

**Authors:** Santiago Navarro-Ledesma, María Aguilar-García, Ana González-Muñoz, Antonio Casas-Barragán, Rosa María Tapia-Haro

**Affiliations:** 1 Department of Physical Therapy, Faculty of Health Sciences, Campus of Melilla, University of Granada, Melilla, Spain; 2 Biomedicine PhD Program, Faculty of Health Sciences, University of Granada, Av. de la Ilustración, 60, 18071 Granada, Spain; 3 Clinical Medicine and Public Health PhD Program, Faculty of Health Sciences, University of Granada, Av. de la Ilustración, 60, 18071 Granada, Spain; 4 Clinica Ana Gonzalez, Malaga, Spain; 5 Department of Physical Therapy, Faculty of Health Sciences, University of Granada (UGR), Instituto de Investigación Biosanitaria ibs.GRANADA, Granada, Spain; 6 Faculty of Health Sciencies, University of Granada (UGR), Ave. de la Ilustración, 60, 18016 Granada, Spain

**Keywords:** Immunology, Neuroscience

## Abstract

Fibromyalgia (FM) is a multicomponent illness and despite its worldwide prevalence, a complete understanding of its aetiology and pathogenesis remains unclear. The goal of the study is to analyze the level of association between elastic properties of tissue measured by strain elastography (SEL) and pain pressure threshold (PPT) in the characteristic painful points described in patients suffering from FM. This was a cross-sectional, observational study. A sample comprised of 42 subjects with FM was recruited from a private care centre. The occiput, low cervical, trapezius, supraspinatus, paraspinous, lateral pectoral, second rib, lateral epicondyle, medial epicondyle, gluteus, greater trochanter, knee, and anterior tibial PPTs were bilaterally assessed using a standard pressure algometer and elastic properties of tissue were evaluated by SEL. Linear regression analysis showed significant associations between SEL and dominant trapezius PPT (*β* = 0.487, 95% CI [0.045, 0.930], *p* = 0.032) after adjustments for the age, body mass index, and menopause status (higher SEL and higher pain sensitivity). No significant associations between SEL and the other PPTs variables were found in women diagnosed with FM. The PPT of the dominant trapezius is associated with SEL measurements in subjects suffering from FM. More studies are required to fully explain the underlying mechanisms.

## Introduction

Fibromyalgia (FM) is characterized as a widespread and diffuse chronic musculoskeletal pain on the human body, accompanying in addition to other symptoms such as dysesthesia, tingling, tender points (TPs), fatigue, morning stiffness, headache, abdominal pain, sleep disturbances, anxiety, altered moods, and concentration difficulties, among others^1–3^. In the worldwide population, between 0.4 and 9.3% of people suffer from FM with an average prevalence rate of 2.7%^4^. The estimated prevalence of FM is 2.31% and 2.40% in the European and Spanish population respectively, with a greater probability in women compared with men (ratio 21:1). Likewise, the frequent age range of clinical manifestations is between 30 and 50 years of age^4,5^.

According to the American College of Rheumatology (ACR), the criteria for diagnosis of FM are: (1) generalised pain for at least 3 months, and (2) TPs on digital palpation, with a force approximately of 4 kg, for at least 11 of the 18 bilateral points^6^. However, in 2016, the ACR included new conditions for diagnosis of a person with FM such as widespread pain for at least 4 of 5 body regions, pain or symptoms maintained during 3 months, or high scores in the Widespread Pain Index (WPI) or Symptom Severity Scale (SSS) scale, indicating a worsening in the widespread and the intensity of pain^2^.

The pathophysiology and underlying mechanisms of FM remain uncertain. Scientific literature considers the phenomenon of central sensitization to be the principal key factor in the development of the FM^7^. However, there are other series of neurogenic, vascular, inflammatory, immunological, nutritional, and genetic factors that could explain this chronic condition syndrome^3,8,9^. In this sense, studies have highlighted the existence of morphological abnormalities in the diameter and density of blood capillaries, which could influence the sympathetic tone and activity and modifying the microcirculatory blood flow, skin conductance, mechanotransduction, and tensegrity of the tissues^10,11^. In this regard, new techniques for the quantification of FM and myofascial pain are appearing, such as magnetic resonance imaging (MRI), microanalytic techniques, infrared thermography and ultrasonography. Among them, ultrasonography is gaining popularity and its use is growing in both research and clinical settings^12^. Specifically, strain elastography (SEL) has the ability to quantify the elasticity of tissue^13^, thus offering possibilities of assessing stiffness and tissue quality, such as in TPs.

A TP is a specific localized area that can be found on a muscle, muscle–tendon junction, fat pad, or bursa region and is extremely sensitive to pressure or touch. These points are frequently linked to musculoskeletal disorders like FM^14–16^.

On the other hand, Myofascial Trigger Points (MTrPs) are described as hyperirritable tenderness points in taut bands of skeletal muscle or their corresponding fascia that cause local pain, referred pain, and a local twitch response when pressure is applied. Clinically, MTsP can be categorized as “latent” or “active”. When mechanically stimulated by palpation or dry needling, an active MTrP produces local and referred pain that the patient can feel. Latent MTrPs are asymptomatic and do not produce palpable pain^14,17^.

A TP, as opposed to a trigger point, should be used to describe an area when local pressure applied to a delicate area causes pain without referred pain or reproduction of pain^14^. It has been established that TP and MTrP can manifest in most muscles of the human body, although some locations, such as the back, face, neck, knee, calf, gluteus maximus, quadriceps, and hips, are most often affected^16,17^.

The elastic characteristics of soft tissues are indicative of alterations in the mechanical functions of soft tissue that occur with age and disease^18^. The mechanical failures that occur in tissues are associated with increases in both morbidity and mortality^18^. Additionally, the elastic properties of soft tissue, as well as cartilaginous tissues, are influenced or even determined by the activity of the sympathetic nervous system^19^. In patients with FM, a relationship between psychological factors and the elasticity of tissues has been shown^20^, as well as with sympathetic nervous system dysfunction^21^, however, little research has been carried out to study elastic properties in tender or MTrp, and the existing results are not yet conclusive. Therefore, there is a need for research to assess such painful points to better understand the relationship between tissue elasticity and pain pressure thresholds (PPT), since SEL can be employed to measure the elastic properties of soft tissue, and a decrease in elasticity indicates the progression of disease and/or the effects of unsuccessful aging^21–23^. We hypothesized that establishing a potential association between the PPT in FM and the elastic properties of soft tissue opens up the possibility of using elastic property measurements to assess treatment effectiveness, particularly when pain and other symptoms, including psychosocial factors, are linked to these properties.

Hence, the aim of the study was to analyse the level of association between SEL and PPT in the characteristic painful points described in patients suffering from FM.

## Methods

### Design

This was a cross-sectional, observational study. This study received ethical approval by the Ethics Committee of Human Research of the University of Granada, Spain (approval number: 1044/CEIH/2020), and was reported in line with the STROBE statement^24^ and conducted by the Declaration of Helsinki. All experiments were performed in accordance with relevant guidelines and regulations.

### Setting

Participants were recruited in a private care centre and rehabilitation service, from January 30 (2021) to January 30 (2022). Formal meetings and trial information sheets were provided to the participants. Informed consent was obtained from all subjects and/or their legal guardian (s).

### Participants

Participants were screened to determine whether they met the following inclusion and exclusion criteria:

#### Inclusion criteria

(i) Aged between 34 and 64 years; ii) FM diagnosis following the ACR classification^2^.

#### Exclusion criteria

(i) Presenting any inflammatory, (ii) orthopedic, (iii) or neurological disease which may affect cognitive, balance, hearing, and vision impairment in terms of the ability to answer questions.

### Recruitment procedures

A physiotherapist collaborating in the project was in contact with the participants at the time of recruitment and carrying out the treatment, as well as providing them the information about the study and eligibility criteria. Those who fit in the study and remained interested in the participation signed a consent form.

### Outcome measures

#### Pain pressure threshold (PPT)

PPT were bilaterally assessed over the 12 initial TPs considered by the ACR for FM diagnosis^6^, together with the anterior tibial, other characteristic painful point presented in central sensitization conditions^25^, with a standard pressure algometer of 1 cm2 (FPK 20; Wagner Instruments, Greenwich, CT, USA).

The TPs assessed were: (i) at the suboccipital muscle insertions, (ii) at the anterior aspects of the intertransverse spaces at low cervical C5–C7, (iii) at the midpoint of the trapezius (upper border), (iv) at the supraspinatus origins, above the scapula spine near the medial border, (v) paraspinous, at the level of the mid-scapula, 3 cm lateral to the midline, (vi) at the second costochondral junctions (second rib), (vii) at the level of the fourth rib at the anterior axillary line (lateral pectoral), (viii) 2 cm distal to the epicondyles, (ix) medial epicondyle, (x) at the distal dorsal third of the forearm, (xi) at the upper outer quadrants of buttocks in the anterior fold of gluteal muscle, (xii) greater trochanter just posterior to the trochanteric prominence and (xiii) at the medial knee fat pad, proximal, to the joint line. We have followed the measurement protocol previously described in literature^26^. We positioned the algometer perpendicular to the TP, to assess PPT, the pain threshold was set as the minimum pressure that caused pain. Positive evidence of a TP was considered to exist when participants reported “pain” at or below a pressure of 4 kg. The mean of three measurements at each TP was used for the analysis. The total count of positive TPs was recorded for each participant^6,26,27^. Pressure algometry is considered a valid and reliable method for the evaluation of pain sensitivity^28^.

#### SEL measurements

**Figure 1:**
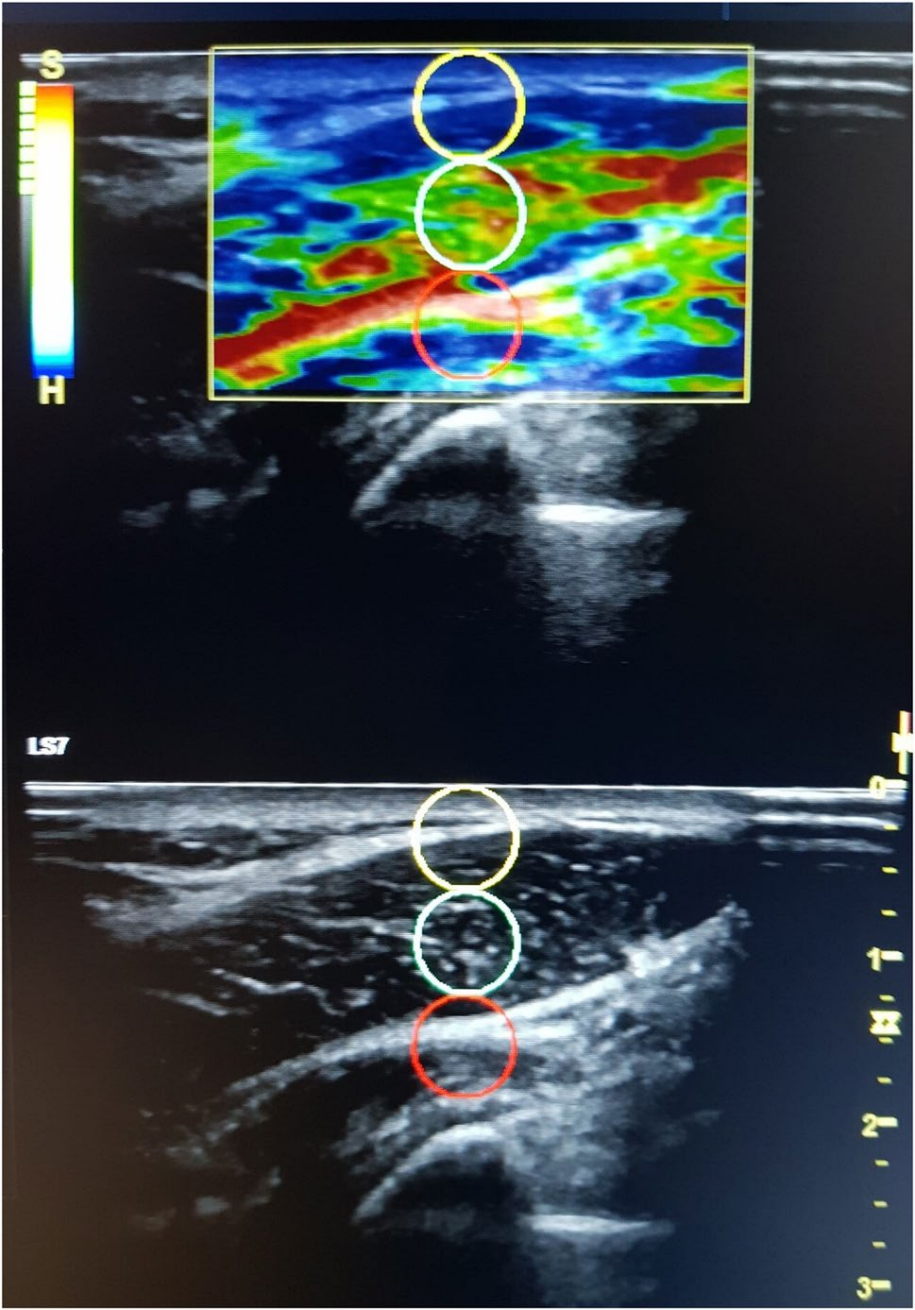
Quantitative strain elastography (SEL) assessment in the upper trapezius muscle in a woman with Fibromyalgia. Different circles represent assessments of different regions of interest (ROI) from superficial to deep tissues.

The Logiq S7 using a 15 MHz linear probe (GE Healthcare, Milwaukee, WI) was used to carry out all the measurements by an expert physiotherapist with eleven years of experience in ultrasound imaging. All participants were positioned in the same orientation used for the MTrPs) identification protocol^29^. All images were obtained with the transducer placed longitudinally with the muscle fibers and positioning the center of the probe over the TP of interest and the control point locations, following recommendations from reliability studies^30^. Then, the tissue was compressed, a software incorporated quality control evaluated the recommended compression size, being approximately 2–5 mm. The exact raw strain value (0–6; with 0 being softest and 6 being the hardest tissue) was calculated using A 5 mm circular region of the area of interest was used to calculate the exact raw strain value, as indicated by the manufacturer’s instructions and following previous studies^26,27^. In order to minimize intra-observer variation, a mean of the three measured areas at each was calculated. Following manufacture’s recommendations, only sequences with the highest image quality were used (Fig. 1).

### Sample size

Sample size was determined using G * Power 3.1.9.7 software (Heinrich-Heine-Universität Düsseldorf, Düsseldorf, Germany). Based on results of PPT through sonoelastograhpy in patients with nonspecific lumbopelvic pain of a previous cross-sectional study^31^, it is necessary to include a minimum of 18 subjects with FM to obtain a power of 80% and an alpha level (α) of 0.05. The sample size was increased to 48 participants after taking into account an expected loss rate of approximately 62.5%. The final sample of the study was 42 women with FM.

### Statistical analysis

The statistical analysis was conducted by SPSS Statistics Version© 24 software for Windows (IBM Corporation, Armonk, NY, USA). The data for continuous and categorical variables were expressed as mean ± standard deviation (SD) and frequency (%), respectively. The normality and distribution of the variables was performed using the Kolmogorov–Smirnov test (*α*-value = 0.05). To test homoscedasticity and independence of the variables, we also performed the Levene test (α-value = 0.05) and Durbin-Watson test. We used a One Sample T-test with 95% confidence interval (95% CI; *α*-value = 0.05) to test the demographic and clinical data. Linear regression analysis was performed to check the associations among SEL and PPTs in FM women after adjustment for age, menopause status, body mass index (BMI). The results of the linear regression analysis were shown as estimate (*β*) with 95% CI and *p*-value. We fixed the level of statistical significance at *p* < 0.05.

### Ethics approval and consent to participate

This study has received ethical approval by the Ethics Committee of Human Research of the University of Granada, Spain (approval number: 1044/CEIH/2020). All participants signed a consent form to participate.

## Results

### Sociodemographic characteristics

**Figure 2:**
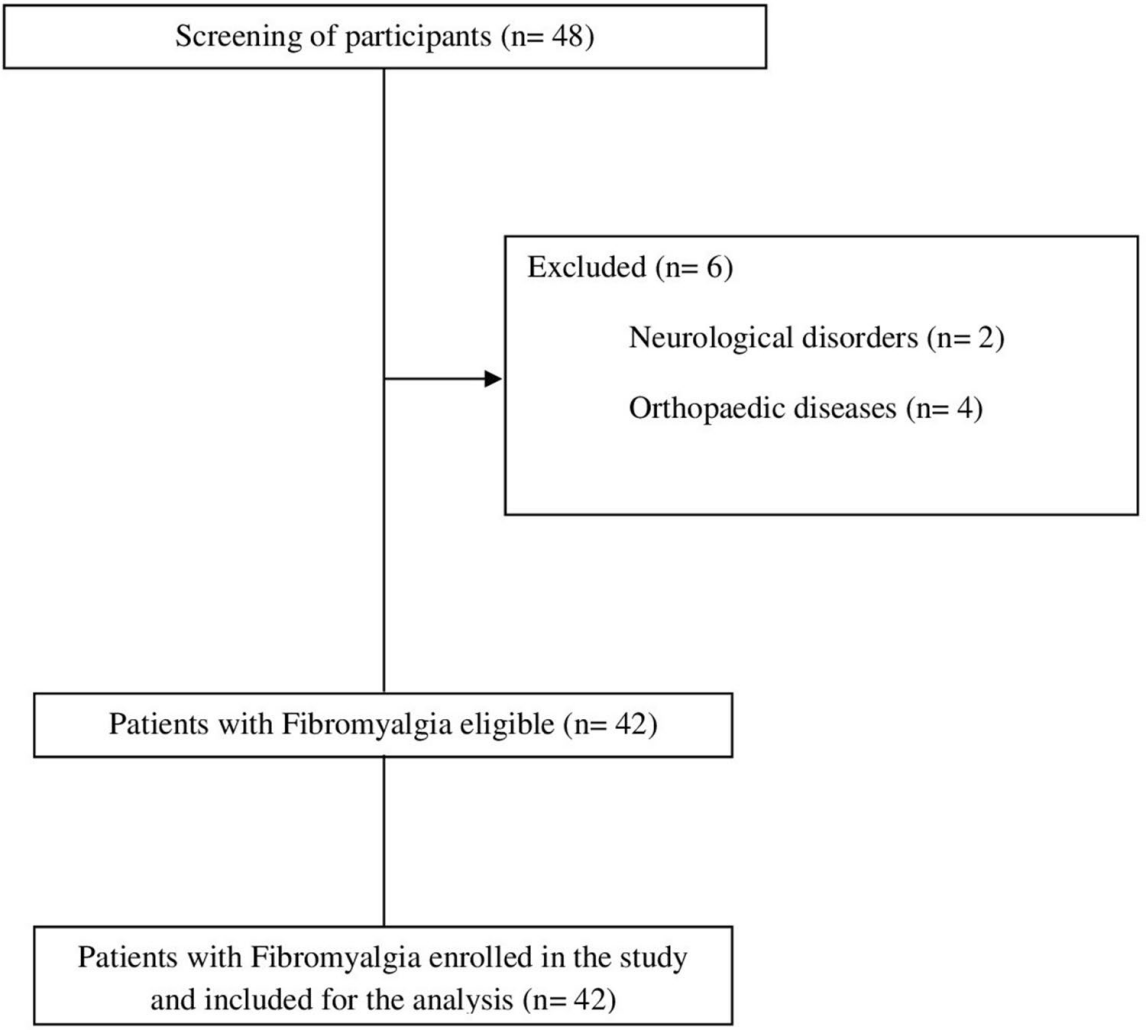
Flow diagram of participants.

Forty-two women with FM (mean age, 52.83 ± 8.04) were recruited and met the inclusion and exclusion criteria established in this study. The sociodemographic data of women with FM are summarised in Table 1. Finally, a flow diagram of study was included (Fig. 2).

**Table 1:** Summary of sociodemographic data and pain pressure threshold of the women with Fibromyalgia.

Variable		Women with Fibromyalgia (n = 42)	
		Mean ± SD/frequency (%)	95% CI
Age (years)		52.83 ± 8.04	[50.33, 55.34]
Height (m)		1.63 ± 0.05	[1.62, 1.65]
Weight (kg)		78.19 ± 18.03	[72.57, 83.81]
BMI (kg/m^2^)		29.32 ± 6.21	[27.38, 31.25]
Menopause status			
Premenopausal		29 (69.05)	
Postmenopausal		13 (30.95)	
Pressure pain thresholds			
Occiput	D	1.54 ± 0.69	[1.32, 1.76]
	ND	1.55 ± 0.87	[1.28, 1.82]
Low cervical	D	1.36 ± 0.56	[1.19, 1.54]
	ND	1.39 ± 0.68	[1.18, 1.60]
Trapezius	D	2.00 ± 0.90	[1.72, 2.29]
	ND	1.69 ± 0.82	[1.44, 1.95]
Supraspinatus	D	2.18 ± 1.09	[1.84, 2.52]
	ND	2.02 ± 0.77	[1.79, 2.27]
Paraspinous	D	2.75 ± 2.18	[2.07, 3.43]
	ND	2.65 ± 1.09	[2.31, 2.99]
Lateral pectoral	D	1.38 ± 0.66	[1.17, 1.57]
	ND	1.82 ± 1.07	[1.48, 2.15]
Second rib	D	0.70 ± 0.97	[0.39, 1.00]
	ND	0.85 ± 1.28	[0.45, 1.25]
Lateral epicondyle	D	1.59 ± 1.36	[1.17, 2.02]
	ND	1.69 ± 1.25	[1.30, 2.08]
Medial epicondyle	D	1.32 ± 0.98	[1.01, 1.62]
	ND	1.45 ± 1.06	[1.12, 1.78]
Gluteus	D	2.14 ± 1.43	[1.70, 2.59]
	ND	1.90 ± 1.27	[1.50, 2.30]
Greater trochanter	D	1.85 ± 1.29	[1.45, 2.26]
	ND	2.01 ± 1.53	[1.54, 2.49]
Knee	D	1.52 ± 1.28	[1.12, 1.92]
	ND	1.50 ± 1.22	[1.12, 1.88]
Anterior Tibial	D	1.76 ± 1.52	[1.29, 2.24]
	ND	1.57 ± 1.14	[1.22, 1.93]

Data are expressed as mean ± SD for quantitative variables and as frequency (%) for qualitative variables.

*SD* standard deviation, *CI* confidence interval, *BMI* body mass index, *D* dominant, *ND* no dominant.

### Associations between strain elastography and pain pressure thresholds in women diagnosed with FM

Table 2 illustrates the associations among SEL and PPT variables in women with FM. Linear analysis regression only revealed that SEL was significantly associated with the dominant trapezius PPT (*β* = 0.487, 95% CI [0.045, 0.930], *p* = 0.032) after adjustment by age, menopause status, and BMI. There were no significant correlations between SEL and the other TPs in the women with FM.

**Table 2:** Correlations between strain elastography (SEL) and pressure pain thresholds (PPTs) in women with Fibromyalgia.

Variable		Women with Fibromyalgia (n = 42)			*p*-value
		Strain elastography (SEL)			
		*Mean* ± *SD*	β	*95% CI*	
Pressure pain thresholds					
Occiput	D	2.42 ± 1.07	0.121	[− 0.435, 0.678]	0.661
	ND	2.58 ± 1.45	0.287	[− 0.350, 0.923]	0.368
Low cervical	D	2.07 ± 0.92	− 0.088	[− 0.640, 0.464]	0.749
	ND	2.17 ± 0.95	− 0.023	[− 0.526, 0.479]	0.926
Trapezius	D	2.34 ± 1.26	0.487	[0.045, 0.930]	0.032*
	ND	2.33 ± 1.27	0.024	[− 0.595, 0.644]	0.937
Supraspinatus	D	2.23 ± 0.88	− 0.104	[− 0.401, 0.192]	0.480
	ND	2.06 ± 0.79	0.121	[− 0.242, 0.485]	0.503
Paraspinous	D	2.82 ± 1.22	− 0.097	[− 0.280, 0.085]	0.287
	ND	2.48 ± 1.14	− 0.041	[− 0.418, 0.336]	0.827
Lateral pectoral	D	1.92 ± 0.77	− 0.034	[− 0.461, 0.394]	0.873
	ND	2.32 ± 0.98	0.171	[− 0.190, 0.531]	0.344
Second rib	D	2.37 ± 0.86	0.146	[− 0.184, 0.476]	0.376
	ND	2.09 ± 0.88	0.127	[− 0.133, 0.387]	0.330
Lateral epicondyle	D	1.79 ± 1.07	− 0.007	[− 0.250, 0.237]	0.957
	ND	1.54 ± 0.88	− 0.003	[− 0.235, 0.229]	0.981
Medial epicondyle	D	1.55 ± 1.00	0.085	[− 0.223, 0.393]	0.580
	ND	1.63 ± 0.99	0.036	[− 0.254, 0.326]	0.804
Gluteus	D	1.72 ± 0.70	− 0.011	[− 0.175, 0.152]	0.888
	ND	1.58 ± 0.75	0.048	[− 0.156, 0.251]	0.638
Greater trochanter	D	1.94 ± 1.23	0.261	[− 0.084, 0.606]	0.134
	ND	1.86 ± 1.11	− 0.094	[− 0.346, 0.158]	0.455
Knee	D	1.62 ± 1.12	0.065	[− 0.227, 0.356]	0.656
	ND	1.56 ± 0.80	− 0.044	[− 0.268, 0.181]	0.697
Anterior Tibial	D	2.42 ± 1.23	− 0.077	[− 0.313, 0.160]	0.515
	ND	2.42 ± 1.10	0.025	[− 0.329, 0.380]	0.886

Beta (*β*) represents the regression coefficient. Adjusted for age, menopause status and body mass index.

*SEL* strain elastography, *SD* standard deviation, *CI* confidence interval, *D* dominant, *ND* no dominant.

*Significance level: *p* < 0.05.

## Discussion

The aim of this study was to analyse the degree of association between the elasticity of the tissues and the level of the PPT in the characteristic TPs established for patients with FM. We hypothesized that SEL would show an increased stiffness measurement in TPs and this would be positively related to the level of the PPT. Our results showed a significant regressions association between SEL measurements and dominant trapezius PPT in patients with FM, but there were no significant regressions between SEL and the rest of the PPTs. Central sensitization is proposed as a primary key factor in the development of FM^7^. Given that the elastic properties of soft tissue are known to be influenced or possibly determined by the activity of the sympathetic nervous system, it is plausible that any dysfunction in the sympathetic nervous system could result in alterations in the tensegrity of the tissue. This, in turn, may significantly impact an individual’s perception of pain^10,11^. It is our hypothesis that if a correlation can be established between PPT and SEL in individuals with FM, it opens up the possibility of using SEL and PPT in the assessment of subjects with FM, and this theory would warrant further investigation. The fact that significant results have been found in the dominant trapezius may indicate that the presented theory only occurs in that point from the 13 TP assessed. Therefore, presenting a painful and stiff dominant trapezius may be a common characteristic and sign in those with FM, which could serve as a part of the explanatory assessment in such condition. However, this is a first research approach, and more studies are needed to corroborate our findings.

The use of SEL as a non-invasive technique to measure the mechanical properties of tissue has been increasing in recent years^13,32,33^. Previous studies have described using SEL in different organs, such as liver fibrosis, breast, thyroid, prostate, kidney and lymph node or low back pain^13,34,35^. In this regard, SEL has been used to analyse the effectiveness of different interventions such as radiofrequency^27,36^;radial extracorporeal shock wave therapy^37^, manual therapy^38^ and conservative physical therapy^39,40^ in diverse pathologies. Additionally, there is a previous study in which SEL was used to evaluate the effects of photobiomodulation on tissues in patients with FM^27^. SEL has also been used to perform a differential diagnosis between patients with psoriasis that suffers chronic widespread pain and patients with FM^41–43^.

To the best of our knowledge, this is the first study assessing the tissue elasticity of the TPs in female patients with FM and the relationship of these TPs to the SEL; therefore, a comparison with other studies is difficult. Muro-Culebras and Cuesta-Vargas evaluated and compared the stiffness of TPs in women with FM and in healthy subjects using sono-myography and sono-myoelastography. They observed hypoechoic areas in the ultrasound images of the upper trapezius in both groups but sono-myoelastographydid not reveal greater stiffness in these areas compared with the rest of the muscle^16^, which is in line with our results. Also, in this regard, a recent study by Karayol and Sibel analysed the level of association between the objectivity of visual analog scale (VAS) values and elasticity values of rhomboid major muscles using Shear Wave Elastography (SWE) in FM and healthy subjects, determining that there was no significant association between VAS scores and SWE values^44^. In line with our results, which show a correlation between the PPT in the trapezius muscle and SEL, the evidence suggests a higher prevalence of MTrPs in the upper quarter muscles^29^, with the trapezius being the most affected muscle in MTrPS^40^.

Recent studies have described that SEL can provide an objective and reproducible measure of the changes in elasticity in MTrPs, showing that stiff nodules vibrate with less amplitude than healthy tissue^39,40,45^. In this context, similarities between TPs and MTsPs have been described, since both are related to pathologies that occurs with non-inflammatory myalgia; TPs in FM and MTrPs in MPS^16^, and are the result of the interaction between the autonomic nervous system and anon-specific response of the central nervous system^46^. In this line, the study by Ge et al. in 2010 support the similarities between TPs and MTrPs, since they found that TPs sites coincide with MTrPs in people with FM^47^.

Since TPs can be associated with a variety of diseases such as infections, inflammation, or lesions and MTrPs are common in patients with a variety of conditions ranging from cancer, neck and shoulder disorders to spinal and musculoskeletal disorders^14,17^, it would be very interesting to carry out future studies that compare TPs between patients with FM and healthy subjects or that analyse and compare the characteristics of TPs/MTrPs in other pathologies.

Tissue quality is understood based on the elastic properties of connective tissue, which means proper loading capacity as well as aging. The muscle elasticity measurement indicates the stiffness of the tissue assessed. The interpretation of a “good” muscle SEL quality assessment would be one showing soft elastic characteristics rather than hard elastic characteristics. This is because of vascularity mainly and sympathetic tone. However, SEL measurements are highly evaluator-dependent and results should be interpreted with caution.

The present study shows strengths that should be highlighted. Firstly, this is the first study that analyses the relationship between the elasticity of tissue and all the PPTs in patients with FM. Secondly, all SEL measurements were carried out by an expert who has more than 10 years of experience in ultrasound and SEL. Finally, this study opens new possibilities of research in relation with soft tissue elasticity and the perception of pain.

### Study limitations

The present study also has some limitations that should be recognized. Sample size is small, with FM participants only, and the design of the study is observational and cross-sectional, which means our results should be interpreted with caution. Besides, SEL measurements, although widely accepted and used, are highly dependent on the operator, which must be taken into account when interpreting our results^48,49^. Future studies should utilise a larger sample size and include a control group in order to corroborate our findings and extrapolate the data.

Furthermore, future studies comparing the results of SEL and PPT in TPs from patients with FM and a healthy population, as well as changes in SEL of the PPTs after a treatment program, are needed.

## Conclusions

In the light of the results, there was an association between SEL measurements and dominant trapezius PPT in subjects with FM, but there was no significant association between SEL and the rest of the PPT points. Further studies to corroborate our findings and to fully explain the underlying mechanism are needed.

## Data Availability

The data will be available by contacting the corresponding author.

## Abbreviations

ACR: American College of Rheumatology
BMI: Body mass index
FM: Fibromyalgia
MRI: Magnetic resonance imaging
MPS: Myofascial pain syndrome
MTrPs: Myofascial trigger points
PPT: Pain pressure thresholds
SEL: Strain elastography
SWE: Shear wave elastography
SSS: Symptom severity scale
TPs: Tender points
WPI: Widespread pain index
